# Etude de l’hémogramme dans la drépanocytose homozygote: à propos de 87 patients

**DOI:** 10.11604/pamj.2016.25.240.11118

**Published:** 2016-12-20

**Authors:** Fatima Dahmani, Souad Benkirane, Jaafar Kouzih, Aziz Woumki, Hassan Mamad, Azlarab Masrar

**Affiliations:** 1 Équipe de Recherche en Hématologie, Laboratoire d’Hématologie, Faculté de Médecine et de Pharmacie, Université Mohammed V, Rabat, Maroc; 2 Laboratoire Central d’Hématologie, Centre Hospitalier Ibn Sina, Rabat, Maroc

**Keywords:** Drépanocytose, hématologie, anémie, Maroc, Sickle cell anemia, hematology, anemia, Morocco

## Abstract

La drépanocytose homozygote, fait partie des hémoglobinopathies les plus fréquentes au Maroc. La drépanocytose est caractérisée par une grande variabilité d’expressions clinique et biologique qui dépendent des facteurs génétiques modulateurs et environnementaux. Elle se manifeste par une anémie régénérative de gravité très variable selon les individus. L’évolution spontanée en l’absence de traitement est le décès précoce. La drépanocytose est caractérisée par une grande variabilité d’expression clinique et biologique qui dépend des facteurs génétiques et environnementaux. Un tableau clinique sévère marqué par une fréquence de transfusion élevée et précoce, des complications infectieuses graves et une mortalité précoce. Un état inflammatoire constant caractérisé par des protéines inflammatoires élevées et état nutritionnel compromis. L’objectif est de déterminer le profil des paramètres hématologiques du drépanocytaire homozygote (SS) marocain au cours des stades stationnaires. Nous avons fait une étude descriptive transversale de 87 patients drépanocytaires (SS). Nous avons réalisé une étude biologique comportant: l’hémogramme avec étude morphologique des globules rouges en coloration MGG et numération automatique des réticulocytes. Les électrophorèses de l’hémoglobine à pH alcalin (8.8) sur gel d’agarose avec intégration densitométrique. L’âge moyen est de 13.22 ans ± 16.36 avec un sex- ratio (H/F) de 1.175 et des extrêmes allant de 0.6 à 36 ans. La répercussion de l’anémie sur le plan biologique, est intense chez 88.5% des patients, 67.8% ont une anémie normocytaire contre 29.9% présentant une microcytose, et 2.3% qui présentaient une macrocytose. Le degré d’anisocytose est lié au degré d’anémie, très évocatrice chez les drépanocytaires homozygotes S/S (95,4%). Une réticulocytose était observée chez nos patients (81,6%) et 52.9% présentaient une thrombocytose. Une leucocytose était observée chez 64.4% des patients et 80.5% ont présenté une neutropénie. Les paramètres de l’hémogramme serviront de base de comparaison lors des crises et permettront d’évaluer l’efficacité de la prise en charge par le clinicien. Les valeurs élevées des globules blancs, plaquettes et CCHM semblent déterminants dans l’expression sévère de la drépanocytose au Maroc. Le profil hématologique du drépanocytaire marocain montre des données semblables à celles rapportées en littérature chez ceux de l’Afrique centrale, avec une leucocytose. Les résultats de notre étude suggèrent que, la drépanocytose est un problème de santé le plus fréquent chez les marocains et que nos résultats sont comparables à ceux décrits dans le syndrome drépanocytaire majeur.

## Abstract (en)

Homozygous sickle cell disease is one of the most frequent haemoglobinopathies in Morocco. Sickle cell disease is characterized by a large clinical and biological expression variability which depends on modulating genetic and environmental factors. Clinical manifestation includes regenerative anemia whose severity may vary among individuals. In the absence of treatment, it results in premature death. Sickle cell disease is characterized by a large clinical and biological expression variability which depends on genetic and environmental factors. A severe clinical picture marked by high early transfusion frequency, severe infectious complications and early mortality. A constant inflammatory condition characterized by elevated inflammatory proteins and compromised nutritional status. The objective of this study is to determine the hematological parameters profile in moroccan patients with homozygous sickle cell (SS) disease during stationary phases. We conducted a cross-sectional descriptive study of 87 patients with sickle cell (SS) disease. We performed a biological study based on: Hemogram with morphological assessment of red blood cells stained with MGG and automated reticulocyte counting; Hemoglobin electrophoresis test performed on alkaline agarose gel (pH 8.8) and densitometric integration. The average age is 13.22 years ± 16.36, ranging betrween 0.6 and 36 years, with a sex ratio (M/F) of 1.175. Biological effects of anemia were intense in 88.5% of patients; 67.8% of patients had normocytic anemia compared with 29.9% with microcytosis, and 2.3% with macrocytosis. The degree of anisocytosis was related to the degree of anemia, very evocative in patients with homozygous S/S (95.4%). Reticulocytosis was observed in 81.6% of patients; 52.9% of patients had thrombocytosis. Leukocytosis was observed in 64.4% of patients; 80.5% of patients had neutropenia. The parameters of the hemogram will serve as a basis for comparison during crises and will make it possible to evaluate the effectiveness of patient management. High white blood cell count, platelets and MCHC seem to be determinant of sickle cell anemia severity in Morocco. The haematological profile of moroccan patients with sickle cell disease exhibits data similar to those reported in literature relating to patients with leucocytosis from Central Africa. The results of our study suggest that sickle cell anemia is the most common health problem in Morocco and they are similar to those for major sickle cell syndrome.

## Introduction

Les hémoglobinopathies sont parmi les maladies héréditaires les plus répandues dans le monde. A l’heure actuelle, près de 5 % de la population mondiale sont porteurs d’un gène responsable d’une anomalie de l’hémoglobine et chaque année, près de 300 000 nourrissons naissent dans le monde avec des syndromes thalassémiques (30 %) ou une drépanocytose (70 %) [1–3]. Cette prévalence des hémoglobinopathies peut atteindre 25% dans certaines régions du monde [2]. La drépanocytose est parmi les maladies monogéniques les plus courantes dans le monde entier [4]. On estime que 312000personnes atteintes de l’hémoglobineSS naissent chaque année dans le monde, avec la majorité de ces naissances 236000 en Afrique sub-saharienne [5]. L’OMS estime le taux des porteurs au Maroc à 6,5%, ce qui laisserait supposer l’existence de 30.000 cas de formes majeures de thalassémie et drépanocytose au Maroc [6]. Plusieurs études menée dans différentes régions du Maroc ont montré que le nord–ouest du Maroc est une zone de prédilection des hémoglobinopathies et que la région de Rabat Salé Kenitra, semble la région la plus touchée plus particulièrement au niveau de Kenitra, au niveau de la commune Mnasra qui constitue un foyer riche d’hémoglobinopathies [7]. Chez ces patients une grande variabilité des données hématologiques est observée selon le génotype, l'âge et le sexe des patients, avec des différences selon que l'examen est réalisé au cours d'une phase stationnaire ou au cours d'une crise ou complication [8, 9]. L'absence d'études locales sur le statut hématologique chez ces patients drépanocytaires âgés de 0.6 à 36 ans en phase stationnaire constitue une difficulté pour une meilleure prise en charge. D'où, l'importance de notre étude qui nous permet d'analyser ces paramètres. L'objectif de la présente étude est d'évaluer les paramètres hématologiques du drépanocytaire homozygote marocain âgé de 0.6 à 36 ans en stade stationnaire dans la région Rabat Salé Kenitra et de les comparer à certains pays de l’Afrique centrale.

## Méthodes

Il s'agit d'une étude descriptive transversale comparative sur les aspects hématologiques du drépanocytaire homozygote marocain âgé de 0.6 à 36 ans en phase stationnaire de la maladie. Nos recherches se sont effectuées au laboratoire central d’hématologie du centre hospitalier Ibn Sina Rabat. La récolte de données s'est déroulée pendant une enquête en juin 2015. La phase stationnaire était définie par l'absence de toute fièvre, de crise vaso-occlusive ou hémolytique. Nos malades sont choisis d’une manière aléatoire dans une population à risque d’hémoglobinopathies de 100 familles. 87 sujets (groupe I) ont été tirés sur la base des critères suivants: sujet drépanocytaire homozygote SS, en phase stationnaire, figurant sur la liste de drépanocytaire recensé, reçu pendant la période de l'étude et âgé de 0.6 à 36ans. Le consentement verbal des parents ou tuteurs était obligatoirement sollicité avant inclusion dans l'étude.

Nous avons considéré comme population de référence 104 sujets de la même population à risque avec hémoglobine AA en bonne santé apparente et âgés de 0.6 à 36ans (groupe II). Pour chaque sujet, les variables hématologiques (hémoglobine, réticulocytes, nombre de globules blancs, nombre de plaquettes, formule leucocytaire), âge et origine géographique ont été recueillies sur une fiche d'enquête.

Chaque sujet a bénéficié d'un prélèvement sanguin de quatre millilitres de sang total dans un tube contenant l'EDTA, après ponction veineuse pour la réalisation d'un hémogramme complet sur automate (Sysmex XE 5000) et l'électrophorèse de l'hémoglobine à pH alcalin sur un équipement à électrophorèse Interlab Pretty.

Nous avons réalisé une étude biologique comportant: l’hémogramme avec étude morphologique des globules rouges en coloration MGG et numération automatique des réticulocytes. Les électrophorèses de l’hémoglobine à pH alcalin (8.8) sur gel d’agarose avec intégration densitométrique.

Cette étude de l’hémogramme permet d’une part, de poser le diagnostic et d’autre part d’en donner les caractéristiques, le volume globulaire moyen (VGM), la concentration corpusculaire moyenne en hémoglobine (CCMH), la teneur corpusculaire moyenne en hémoglobine (TCMH). Ainsi que les caractéristiques de la ligne leucocytaire. L’anémie était définie par un taux d’hémoglobine (Hb) inférieur à 11 g/dl et typée, selon le volume globulaire moyen (VGM), en anémie macrocytaire (VGM > 90 FL), normocytaire (80 FL≤VGM≤90 FL) et microcytaire (VGM < 80 FL). En effet, selon la teneur corpusculaire moyenne en hémoglobine (TCMH) dont la valeur normale varie de 32 à 36 picogrammes, on distingue les anémies hypochromes (TCMH < 32 picogrammes) et les anémies normochromes (36 < TCMH > 32 picogrammes). Elle était considérée comme sévère si Hb était inférieur à 7 g/dl, modérée si 7 g/dl≤ Hb<9 g/dl et légère si 9 g/dl≤ Hb< 11 g/dl.

**Interrogatoire:** pour chaque patient, nous avons recueillies les renseignements démographiques, à savoir l'âge, le sexe, l'origine géographique, les antécédents personnels et/ou familiaux, et d'éventuels notions de consanguinité.

**Exploration hématologique:** les critères diagnostics appliqués dans cette étude sont un hémogramme anormal, frottis sanguin présentant des anomalies érythrocytaires et un profil électrophorétique correspondant à une hémoglobinopathieSS.

**Etape pré-analytique:** les prélèvements ont été effectués, pour chacun des patients de notre étude, sur tube anticoagulé (EDTA) pour l’hémogramme et l'étude de l’hémoglobine.

**Etape analytique:** l’hémogramme a été réalisé sur automate Sysmex XE 5000. L’électrophorèse de l'hémoglobine à pH alcalin sur un équipement à électrophorèse Interlab Pretty. Les résultats sont donnés sous forme de tableaux. La différence a été considérée significative pour la valeur p ≤ 0,05. Nous avons utilisé les tests de statistiques descriptives qui nous ont permis de calculer la moyenne et l’écart-type.

**Considérations éthiques:** un consentement libre et éclairé (verbal ou écrit) de toutes les personnes impliquées dans cette étude a été obtenu au préalable.

## Résultats

### Aspects épidémiologiques

87 patients présentant une drépanocytose homozygote. 47 Patients étaient de sexe masculin et 40 de sexe féminin chez les SS avec un sex-ratio (H/F) de 1.175. L’âge moyen des patients est de 13.22ans ± 16.36 et des extrêmes de 0.6 à 36 ans. La distribution des malades par tranche d’âge dégage quatre classes différentes (Tableau 1).

**Tableau 1:** La distribution des malades par tranche d’âge

Caractéristiques	Effectif N	Pourcentage (%)
**Age des patients (ans)**		
0-5 ans	32	36.8%
6-10 ans	22	25.3 %
11-16 ans	19	21.8 %
>16 ans	14	16.1%
**Sexe**		
Masculin	47	54%
Féminin	40	46%

### Aspects biologiques

Les résultats obtenus permettent de constater une diminution statistiquement significative du taux d’hémoglobine (P <0,0001) avec une moyenne de (7.59 ± 0.9181) par apport aux témoins (12.06± 2.04). Nos résultats révèlent une diminution non significative (P=0.63) de la teneur corpusculaire moyenne en hémoglobine chez ces malades (28.02±4,50) par apport aux témoins (25.38±4,42). Les résultats obtenus permettent de constater une diminution du volume globulaire moyen (p= 0.04) chez le groupe malade et une moyenne de (80.81±12.37) par apport aux témoins (74.31±10.91). Concernant le pourcentage de réticulocytes il a varié autour d'une moyenne de 363 ± 195,5 (G/L) dans le groupe I contre une moyenne de 57 ± 41(G/L) dans le groupe II. La comparaison de ces moyennes donne une différence statistiquement significat ive (P<0,0001). Une anémie est observée chez 88.5% des patients SS. Le degré d’anémie est très variable, (78.2%) des malades présentant une anémie normochrome. (67.8%) ont une anémie normocytaire contre (29.9%) présentant une microcytose, on avait (2.3%) qui présentent une macrocytose. Le RDW avec une moyenne de 19.34± 4.47%contre une moyenne de 15.34±2.85% dans le groupe II, La comparaison de ces moyennes donne une différence statistiquement significative (P<0,003). Le degré d’anisocytose est lié au degré d’anémie, très évocatrice chez les drépanocytaires homozygotes S/S (95,4%)avec un (P<0,003).

Le taux de plaquettes dans le groupe I avec une moyenne de 335.90± 101.11giga/L contre une moyenne de 291.42±101.09 giga/L dans le groupe II. La comparaison des deux moyennes ne montre pas de différence significat ive (p=0,123).Nos malades présentaient une thrombocytose avec (52.9%).

Quant au taux de globules blancs, la moyenne est de 13.55±5.32 giga/L dans le groupe I alors qu'elle est de 8.25±2,96 giga/L dans le groupe II. L'analyse statistique montre une différence significative quant à la comparaison de ces deux moyennes (p=0,002). Nous avons observé des différences statistiquement significatives en fonction des éléments de la formule leucocytaire. Neutrophiles: moyennes respectivement de 5.02±1.86 (giga/L) et 3.93± 2.04 dans les groupes I et II (p=0,05).La leucocytose était observée chez (64.4%), (80.5%) des malades ont présenté une neutropénie. Le Tableau 2 rapporte les valeurs moyennes des paramètres hématologiques des malades.

**Tableau 2:** Paramètres hématologiques des drépanocytaires SS et du groupe de référence

Paramètre	Groupe hétérozygotie composite S/ S (n=87)	Groupe témoin (n=104)	p
Hémoglobine (g/dL)	7.59 ± 0.9181	12.06± 2.04	<0,0001
VGM (μm3)	80.81± 12.3708	74.31± 10.91	0,04
TCMH (pg)	28.0273 ± 4.5076	25.38± 4.42	0,63
CCMH (%)	34.6545± 1.0463	33.79± 1.88	0,19
Réticulocytes (G/L	363 ± 195,5	57 ± 41	<0,0001
Plaquettes (giga/L)	335.90± 101,11	291.42± 101.09	0,123
Leucocytes (giga/L)	13.5573± 5.32	8.25± 2.96	0,002
Neutrophiles (giga/L)	5.0282 ± 1,86	3.93 ± 2.04	0,05
RDW (%)	19.3455± 4,47	15.34 ±2.85	0,003

*VGM*: Volume Globulaire Moyen ; *TCMH*: Teneur Corpusculaire Moyenne en Hémoglobine; *C.C.M.H* : concentration corpusculaire moyenne en hémoglobine. *RDW* : indice de distribution érythrocytaire (redcell distribution width).

## Discussion

Dans la présente étude réalisée au laboratoire central d’hématologie de l’Hôpital IBN SINA de Rabat. La population étudiée est constituée de 40 (46%) de sexe féminin et de 47 (54%) de sexe masculin avec un sex-ratio (H/F) de 1.175, Il n'y aurait donc pas de lien significatif entre le type d'hémoglobine et le sexe, ceci a une base génétique car la transmission de la tare se fait indépendamment du sexe. Il s’agit des mêmes observations qui ont été faites par Diagne et Mabiala [10, 11].

L’âge moyen des patients est de 13.22ans ± 16.36 et des extrêmes allant de 0.6 à 36 ans. Notre population d'étude était particulièrement jeune, 83.9 % de l'échantillon avait moins de 16 ans. On remarque que le pourcentage des sujets drépanocytaires homozygotes diminue avec l’âge ceci peut être expliqué par la mortalité précoce de ces sujets les données de l’enquête font ressortir une mortalité infantile, au sein des familles à risque, il est certain que ces enfants morts étaient porteurs d‘hémoglobine SS. Selon certains auteurs 95%des enfants SS mourraient avant l'âge de 15 ans [12]. Cependant d'autres auteurs ont rapportés plusieurs séries de drépanocytaires homozygotes parvenus à l'âge adulte. BARBUTIN-LARRIEU avance 10 à 50 % de survie à 20 ans selon les pays [13].

Dès l'âge de 6 mois, la disparition de l'hémoglobine fœtale entraîne l'apparition des manifestations cliniques justifiant une pratique plus précoce de l'électrophorèse. D’après Bernard et al, 1998 [14], la drépanocytose homozygote est une maladie très grave qui se révélée dés la petite enfance par une altération générale, et une anémie hémolytique chronique débutant dés le sixième mois de vie d’un enfant présentant une splénomégalie [15, 16]. Elle est mortelle avant 20 ans dans la majorité des cas [14].

Les cas étudiés sont tous originaires de la région Rabat Salé Kenitra qui est la région la plus touchée au Maroc, anciennement impaludée, ce dernier exerce une pression sélective par procuration d’un avantage de survie aux porteurs des hémoglobinopathies [17, 18], ceci peut expliquer le succès de la mutation drépanocytaire. Cette région se singularise aussi par un très haut degré de consanguinité. Le Maroc reste un pays très enraciné dans ses traditions et coutumes, et l’endogamie en est la preuve

Les résultats obtenus montrent qu’il existe une diminution très hautement significative (p < 0, 0001) du taux de l’hémoglobine chez ces malades (7.59 ± 0.9181) par apport aux témoins (12.06± 2.04). Mick Ya PongomboShongo et al [19] ont rapporté 8,33±1,35 g/dLpour la forme SS.

Selon Kafando et al [20]; le remplacement par une valine de l’acide glutamique en position 6 de la chaîne de la globine entraine la production anormale de l’hémoglobine S (Hb S), ce qui cause la déshydratation des globules rouges et un défaut de leur déformabilité (drépanocytes) liées à la polymérisation des molécules d’HbS en milieu pauvre en oxygène. Les examens biologiques servent alors à surveiller l‘état basal: numération formule sanguine et réticulocytes avec anémie hémolytique régénérative, hyperleucocytose et hyperplaquettose.

On remarque des anomalies fonctionnelles de la membrane accompagnent la falciformation. Tostson [21] a été le premier à montrer l’augmentation de la perméabilité passive des cations monovalents Na+ et K+au cours de la désoxygénation de courte durée des globules drépanocytaires, sans modification du contenu en eau ni de la quantité totale de cations monovalents. Il est suggéré que l’hémoglobine polymérisée modifié l’interaction du cytosquelette et de la membrane en altérant ce réseau. L’asymétrie des lipides de la membrane est perdue en raison de la mobilité accrue des phospholipides entre les deux couches lipidiques.

Dans notre étude, tous nos patients étaient anémiés, il s'agissait d'une anémie modérée (88.5%). Les anémies microcytaires représentent à peu près un tiers des anémies diagnostiquées (29.9%), alors que les anémies normocytaires représentent les deux tiers (67.8%). L’anémie microcytaires se voit chez les deux sexes. Les anémies observées dans notre étude étaient principalement de type normochrome normocytaire et normochrome microcytaire. Ces résultats sont en concordance avec les valeurs de la littérature qui donne principalement une anémie de type normochrome normocytaire au cours de cette affection. [19, 22, 23].

Selon Vanbourdolle et al [16]; la solubilité de la forme oxygénée de l’hémoglobine S est identique à celle de l’hémoglobine normale. Alors cette hémoglobine S sous forme désoxygénée est moins soluble que l’hémoglobine A. Elle peut se polymériser et permettre la rigidification, l’agglutination et la falciformation des hématies. Il se produit une hémolyse exagérée qui entraine une anémie.

La macrocytose observée pourrait s'expliquer par la présence d'une hyperhémolyse qui a eu pour conséquence une diminution du taux d'Hb et une stimulation de l'érythropoïèse avec production de réticulocytes dont le VGM est plus élevé que celui des érythrocytes [24].

L’hémolyse chronique induit une forte régénération médullaire pour compenser l'anémie ce qui entraîne une carence en acide folique responsable de la macrocytose. Mais même si le traitement en acide folique a été institué pour palier aux carences et corriger l'anémie chez la plupart des patients SS, nous ne pouvons attester de l'observance puis que le traitement était ambulatoire.

Le taux de plaquettes dans le groupe I avait une moyenne de 335.90± 101.11giga/L contre une moyenne de 291.42±101.09 giga/L dans le groupe II. La comparaison des deux moyennes ne montre pas de différence significat ive (p=0,123).

Ce résultat est en accord avec les études de Jaffe à Ontario au Canada et Osaghae à Benin city au Nigeria, qui montrent que la numération plaquettaire est supérieure en cas de drépanocytose comparativement à la population de référence [25, 26]. Les plaquettes activées sécrètent la thrombospondine (TSP) impliquée dans le pontage GR-endothélium et participent à l’état d'hypercoagulabilité de la maladie drépanocytaire contribuant ainsi à la survenue des crises [27].

En ce qui concerne la lignée leucocytaire une différence significative a été notée pour le nombre des polynucléaires neutrophiles (p=0,05), 64.4% des malades soufrent de leucocytose, 80.5% de neutropénie.

L'hyperleucocytose notée chez les homozygotes pourrait être expliquée par le mécanisme d'une hyper production médullaire associée à une démarginalisation permanente du pool marginal vers le pool circulant [28]. D'ailleurs l'hyperleucocytose ne fait partie ni des signes hématologiques des autres anémies hémolytiques, ni de ceux des splénectomisés en dehors de la drépanocytose [28]. La drépanocytose est en effet une maladie inflammatoire dont l’un des marqueurs est la leucocytose [29]. Ce trouble paraît donc être un signe spécifique de cette hémoglobinopathie puisque les patients présentant les signes d'une éventuelle infection ont été diminués, aussi L'absence d'hyperalpha-2 globulines à l'électrophorèse standard sur gel d'agarose exclu la présence d'une réaction inflammatoire susceptible d'augmenter également le nombre de leucocyte.

L’augmentation du nombre et l’activation des leucocytes sont des médiateurs importants de l’inflammation dans la drépanocytose.

Chez ces malades, un état sévère est marqué par une fréquence de transfusion élevée et précoce. En effet les transfusions répétées exposent les patients drépanocytaires à de nombreux risques tels que la transmission d’infection, l’allo-immunisation ou encore la surcharge en fer. On remarque un état inflammatoire constant caractérisé par des protéines inflammatoires élevées et un état nutritionnel compromis [27], des réactions immunologiques variées peuvent survenir, dans n’importe quel contexte de transfusions [30–32].

Les valeurs élevées des Globules Blancs, réticulocytes et CCHM semblent déterminants dans l’expression sévère de la drépanocytose au Maroc [19]. Le profil hématologique du drépanocytaire marocain montre des données semblables à celles rapportées en littérature.

### Hémogramme du drépanocytaire marocain comparé aux hémogrammes des pays de l’Afrique centrale

Notre étude comparé à une étude faite en 2013 [33] sur quatre séries de patients drépanocytaires (SS) dont la première avec 167 sujets congolais d’âge moyen de 12 ans, la deuxième avec 299 sujets gabonais d’âge moyen de 12,2 ans, la troisième avec 57 sujets camerounais d’âge moyen de 12,3 ans, la quatrième série avec 47 sujets tanzaniens d’âge moyen de 5,1. Qui ont fait l’objet de l’analyse de l’hémogramme au cours des stades stationnaires.

L’hémogramme des pays de l’Afrique centrale a montré les valeurs moyennes suivantes: Hb: 7,4g/dl; CCMH: 32,4pg; VGM: 83,4 f/l; GB: 16,7G/L; plaquettes: 379,8. La moyenne de l’Hb totale chez le drépanocytaire de l’Afrique centrale était pratiquement semblable à celle des marocains de la même tranche d’âge, le taux moyen du VGM des africains était presque identique à celui des marocains, le taux de GB chez les africains était peu élevé comparé aux marocains, le taux de plaquettes était le même chez les pays de l’Afrique centrale comparés aux marocains (Tableau 3). On a trouvé que le drépanocytaire homozygote marocain présente un peu prés le même profil hématologique que le drépanocytaire de l’Afrique centrale (Tableau 3).

**Tableau 3:** Hémogramme du drépanocytaire marocain comparé aux hémogrammes des pays africains

Pays	Nombre	Age	HB	CCMH	GB	VGM	Plaquettes
Gabon	299	12	7.4	32.4	16.9	79.5	398
Congo	167	11.4	7.3	32.2	16.5	89.9	344
Cameroun	57	12.3	7.8	32.1	17	81.1	388
Tanzanie	47	5.1	7.2	33.3	16.7	86.9	377
Maroc	87	13	7.59	34.6	13.55	80.81	336

Les résultats de notre étude rencontrent ce qui est souvent décrit dans le syndrome drépanocytaire majeur sur les homozygotes SS. Alors il faut noter que: la drépanocytose est caractérisée par une grande variabilité d’expression clinique et biologique qui dépend des facteurs génétiques et environnementaux; un tableau clinique sévère marqué par une fréquence de transfusion élevée et précoce, des complications infectieuses graves et une mortalité précoce; un état inflammatoire constant caractérisé par des protéines inflammatoires élevées et état nutritionnel compromis.

## Conclusion

Nos résultats sont comparables à ceux souvent décrits dans le syndrome drépanocytaire majeur. La drépanocytose reste un véritable problème de santé publique dans notre pays. Faute de dépistage néonatal systématique, le diagnostic est souvent posé en présence d'un signe d'appel. La meilleure connaissance des différents aspects biologiques de la maladie devraient permettre de réduire la mortalité chez les homozygotes SS. Les paramètres de l’hémogramme serviront de base de comparaison lors des crises et permettront d’évaluer l’efficacité de la prise en charge par le clinicien. Les valeurs élevées des globules blancs, plaquettes et CCHM semblent déterminants dans l’expression sévère de la drépanocytose au Maroc. Le profil hématologique du drépanocytaire marocain montre des données semblables à celles rapportées en littérature chez ceux de l’Afrique centrale, avec une leucocytose. La drépanocytose a besoin de stratégies, plus de sensibilisation du public et de prévention dans notre pays. Les programmes d'éducation et de dépistage prénatal obligatoire semblent être à l'ordre du jour.

### Etat des connaissances actuelles sur le sujet

- La drépanocytose est caractérisée par une grande variabilité d’expression clinique et biologique qui dépend des facteurs génétiques et environnementaux;
- Un tableau clinique sévère marqué par une fréquence de transfusion élevée et précoce, des complications infectieuses graves et une mortalité précoce;
- Un état inflammatoire constant caractérisé par des protéines inflammatoires élevées et état nutritionnel compromis.

### Contribution de notre étude à la connaissance

- Les paramètres de l’hémogramme serviront de base de comparaison lors des crises et permettront d’évaluer l’efficacité de la prise en charge par le clinicien;
- Les valeurs élevées des globules blancs, plaquettes et CCHM semblent déterminants dans l’expression sévère de la drépanocytose au Maroc;
- Le profil hématologique du drépanocytaire marocain montre des données semblables à celles rapportées en littérature chez ceux de l’Afrique centrale, avec une leucocytose.
